# Phylogenomic analyses and host range prediction of cluster P mycobacteriophages

**DOI:** 10.1093/g3journal/jkac244

**Published:** 2022-09-12

**Authors:** Abigail A Howell, Cyril J Versoza, Gabriella Cerna, Tyler Johnston, Shriya Kakde, Keith Karuku, Maria Kowal, Jasmine Monahan, Jillian Murray, Teresa Nguyen, Aurely Sanchez Carreon, Abigail Streiff, Blake Su, Faith Youkhana, Saige Munig, Zeel Patel, Minerva So, Makena Sy, Sarah Weiss, Susanne P Pfeifer

**Affiliations:** School of Life Sciences, Arizona State University, Tempe, AZ 85281, USA; Biodesign Institute, Arizona State University, Tempe, AZ 85281, USA; School of Life Sciences, Arizona State University, Tempe, AZ 85281, USA; Center for Evolution and Medicine, Arizona State University, Tempe, AZ 85281, USA; School of Life Sciences, Arizona State University, Tempe, AZ 85281, USA; Biodesign Institute, Arizona State University, Tempe, AZ 85281, USA; School of Molecular Sciences, Arizona State University, Tempe, AZ 85281, USA; School of Molecular Sciences, Arizona State University, Tempe, AZ 85281, USA; School of Life Sciences, Arizona State University, Tempe, AZ 85281, USA; School of Life Sciences, Arizona State University, Tempe, AZ 85281, USA; School of Life Sciences, Arizona State University, Tempe, AZ 85281, USA; School of Life Sciences, Arizona State University, Tempe, AZ 85281, USA; School of Life Sciences, Arizona State University, Tempe, AZ 85281, USA; School of Molecular Sciences, Arizona State University, Tempe, AZ 85281, USA; School of Life Sciences, Arizona State University, Tempe, AZ 85281, USA; School of Life Sciences, Arizona State University, Tempe, AZ 85281, USA; Biodesign Institute, Arizona State University, Tempe, AZ 85281, USA; School of Molecular Sciences, Arizona State University, Tempe, AZ 85281, USA; School of Molecular Sciences, Arizona State University, Tempe, AZ 85281, USA; School of Life Sciences, Arizona State University, Tempe, AZ 85281, USA; School of Politics and Global Studies, Arizona State University, Tempe, AZ 85281, USA; School of Molecular Sciences, Arizona State University, Tempe, AZ 85281, USA; School of Life Sciences, Arizona State University, Tempe, AZ 85281, USA; School of Life Sciences, Arizona State University, Tempe, AZ 85281, USA; School of Life Sciences, Arizona State University, Tempe, AZ 85281, USA; School of Life Sciences, Arizona State University, Tempe, AZ 85281, USA; School of Life Sciences, Arizona State University, Tempe, AZ 85281, USA; School of Life Sciences, Arizona State University, Tempe, AZ 85281, USA; Center for Evolution and Medicine, Arizona State University, Tempe, AZ 85281, USA

**Keywords:** mycobacteriophages, cluster P, phylogenomics, comparative genomics, host range

## Abstract

Bacteriophages, infecting bacterial hosts in every environment on our planet, are a driver of adaptive evolution in bacterial communities. At the same time, the host range of many bacteriophages—and thus one of the selective pressures acting on complex microbial systems in nature—remains poorly characterized. Here, we computationally inferred the putative host ranges of 40 cluster P mycobacteriophages, including members from 6 subclusters (P1–P6). A series of comparative genomic analyses revealed that mycobacteriophages of subcluster P1 are restricted to the *Mycobacterium* genus, whereas mycobacteriophages of subclusters P2–P6 are likely also able to infect other genera, several of which are commonly associated with human disease. Further genomic analysis highlighted that the majority of cluster P mycobacteriophages harbor a conserved integration-dependent immunity system, hypothesized to be the ancestral state of a genetic switch that controls the shift between lytic and lysogenic life cycles—a temperate characteristic that impedes their usage in antibacterial applications.

## Introduction

Less than 1% of the virosphere on our planet has been characterized to date (Geoghegan and Holmes 2017). An important part of this virosphere is bacteriophages (i.e. bacteria-infecting viruses), which are impacting bacterial genome evolution and community dynamics in every environment (Howard-Varona *et al.* 2017).

Bacteriophages can establish lytic or lysogenic infections—the former leading to cell destruction while the latter being “dormant,” with bacteriophages replicating as prophages within the host without the production of virions (Howard-Varona *et al.* 2017). Temperate bacteriophages can switch between lytic and lysogenic life cycles, for example through the usage of integration-dependent immunity systems that establish lysogeny by suppressing lytic growth through an interplay between 3 proteins: integrase (Int), repressor (Rep), and Cro [for an in-depth discussion on these and other genetic switches, see the commentary by Broussard and Hatfull (2013)]. In integration-dependent immunity systems, the decision on whether lytic or lysogenic growth will take place depends by and large on the activity of Int as modulated by targeted proteolysis (Broussard *et al.* 2013). Under conditions where integrases are broken down (i.e. in the presence of a C-terminal ssrA-like protease degradation tag in Int), integration fails to occur. Instead, the viral form of Rep is generated and subsequently degraded due to the presence of its own C-terminal ssrA-like tag. The lytic protein Cro is freely expressed and stops repressor function (Hochschild *et al.* 1986). Conversely, when integrases escape proteolysis due to either decreased levels of proteases (such as ClpXP) or high multiplicity of infection (i.e. a high ratio of bacteriophages to infection targets), integration of bacteriophage genetic material will occur. This leads to the expression of an active (truncated) form of Rep that lacks the ssrA-like tag, causing a downregulation of Cro expression, which ultimately leads to lysogenic establishment and prophage induction. Thereby, the integration into the host genome is mediated by recombination between the bacteriophage attachment site (*attP*) and the bacterial attachment site (*attB*) in the host genome. Attachment sites are recognized by Int—an integral part of the *attP*–*Int* cassette required for integrase-mediated site-specific recombination (Singh *et al.* 2013). Thereby, Int is either a tyrosine recombinase (which requires additional host cofactors such as the one present in *Mycobacterium smegmatis*; Pedulla *et al.* 1996; Peña *et al.* 1999; Lewis and Hatfull 2003; Chen *et al.* 2019) or a serine recombinase (which functions without any cofactors but recognizes shorter *attP* sequences than the tyrosine recombinase; Groth and Calos 2004).

Mycobacteriophages are a group of both lytic and temperate bacteriophages that infect mycobacterial hosts—including the causative agents for several human diseases such as tuberculosis (*M. tuberculosis*) or leprosy (*M. leprae*), separated into 31 clusters (A–Z and AA–AE) based on their nucleotide similarity and genomic architecture (Pope *et al.* 2011). Out of these, temperate cluster P bacteriophages are of particular interest to the scientific community to, for example study the evolution of genetic switches as several members of this cluster have been shown to harbor an unusual switch in which the bacteriophage attachment site is located within the repressor gene (e.g. Broussard *et al.* 2013; Doyle *et al.* 2017).

Interestingly, many mycobacteriophages have the ability to broaden their host range to infect either different strains or completely new mycobacterial species (Jacobs-Sera *et al.* 2012). In contrast to lytic bacteriophages, which are frequently exploited as antimicrobial agents (Sharma *et al.* 2017), the life cycle of temperate bacteriophages often impedes their usage, particularly with regard to bacteriophage therapy, due to the risk of transferring virulence factors through genomic pathogenicity islands (Malachowa and Deleo 2010; Xia and Wolz 2014). Thus, host ranges of many temperate bacteriophages remain poorly characterized, despite their important impact on bacterial evolution. To advance our knowledge on the topic, and as part of a course-based undergraduate research experience at Arizona State University, we analyzed the genomes and computationally inferred the host ranges of 40 cluster P mycobacteriophages.

## Materials and methods

### Comparative genomic analyses

A multiple sequence alignment of 40 cluster P mycobacteriophages previously isolated in *M. smegmatis* mc^2^155 (Supplementary Table 1) was generated via MAFFT v.7.407 (Katoh and Standley 2013) and subsequently used to construct a neighbor-joining tree in MEGA X (Kumar *et al.* 2018) using a bootstrap test of phylogeny with 10,000 replicates. Additional whole-genome and gene-specific trees were generated, including 16 bacteriophages from clusters G1, I1, and N for which integration-dependent immunity systems had previously been identified (either experimentally or through the computational identification of an *attP* site within the repressor gene; Supplementary Table 2). Trees were visualized using FigTree v.1.4.4 (http://tree.bio.ed.ac.uk/software/figtree/; last accessed 2022 April 24) and the Interactive Tree Of Life (Letunic and Bork 2019). Sequence relatedness was determined using pairwise average nucleotide identity scores calculated using the DNA Master “Genome Comparison” tool v.5.23.6 and plotted using the ggplot2 function (Wickham 2016) in R v.4.0.2. All software were executed using default settings.

### Identification of *attP* and *attB* sites

Following Pham *et al.* (2007), NCBI BLASTn (Altschul *et al.* 1990) was used to compare the 300-bp region surrounding the 5′-end of the immunity repressor gene in each cluster P mycobacteriophage (Supplementary Table 1) against the genomes of 14 putative mycobacterial host species (Supplementary Table 3) to determine the plausibility of *attP*/*attB* sites. In addition, Tandem Repeats Finder v.4.09 (Benson 1999) was used to search for integrase binding sites near the *attP* common core.

### Host prediction

Following the best practices suggested by Versoza and Pfeifer (2022), both exploratory and confirmatory methods were used to computationally predict host ranges for 40 closely related cluster P mycobacteriophages (Supplementary Table 1). First, the exploratory tool PHERI v.0.2 (Baláž *et al.* 2020) was used to predict bacterial host genera. Among the currently available exploratory host range prediction tools, PHERI was the most user-friendly and well-documented, making it ideally suited for course-based undergraduate research experiences. Next, WIsH v.1.1 (Galiez *et al.* 2017)—a bacterial host range predictor that compares virus and host sequence composition—was used to estimate the likelihood of these 40 cluster P bacteriophages to infect 14 putative mycobacterial host species with particular relevance to human health and disease (Supplementary Table 3). WIsH was selected as the representative for confirmatory host range prediction tools as it was an easily applicable alternative to alignment-based tools which frequently underpredict phage–host interactions (Zielezinski *et al.* 2021). Lastly, following Crane *et al.* (2021), PHASTER (Arndt *et al.* 2016) was used to search the genome of these putative host species for prophages to determine whether cluster P mycobacteriophages might be able to integrate into the host.

## Results and discussion

**Fig. 1.**
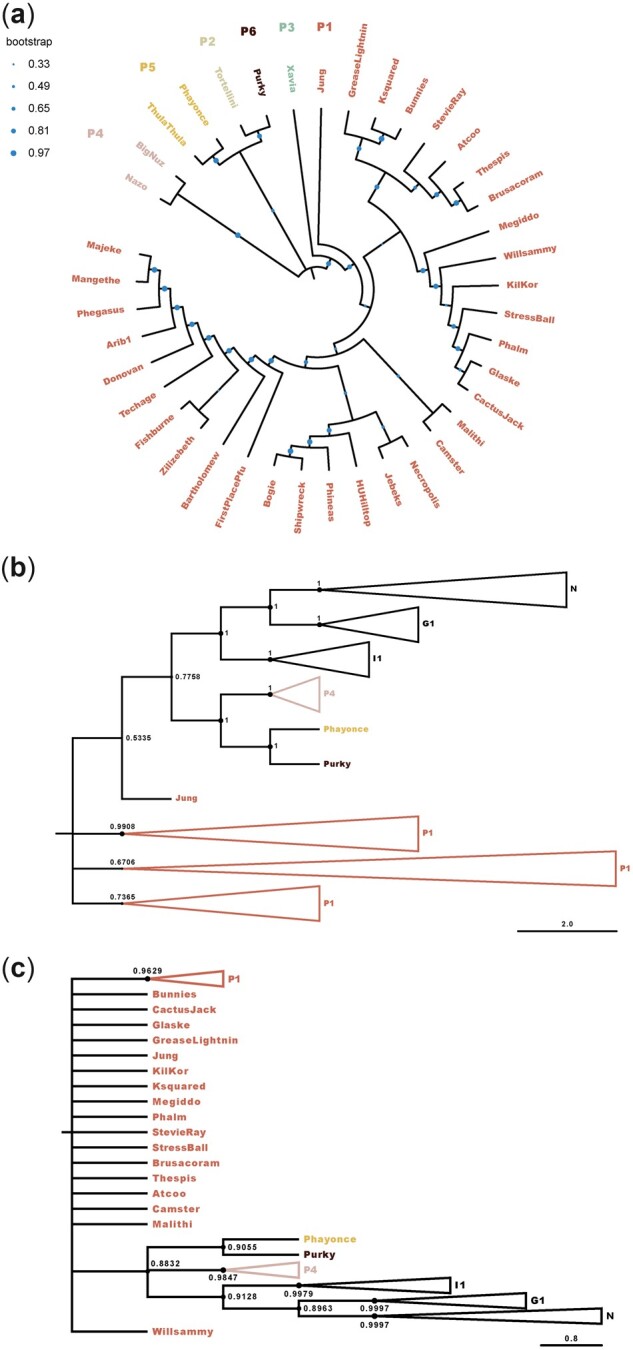
Neighbor-joining trees. Neighbor-joining trees generated in MAFFT (Katoh and Standley 2013) using the multiple-sequence alignment of (a) 40 cluster P mycobacteriophages (Supplementary Table 1) and (b) 16 cluster G1, I1, and N bacteriophages with a previously identified integration-dependent immunity system (Supplementary Table 2), with 10,000 bootstrap replicates. c) Gene-specific tree based on the immunity repressor sequences of the bacteriophages included in (b). Colors highlight membership in subclusters P1–P6.

Comparative genomic analyses between 40 cluster P mycobacteriophages (32 subcluster P1, 1 subcluster P2, 1 subcluster P3, 2 subcluster P4, 2 subcluster P5, and 1 subcluster P6; Supplementary Table 1) demonstrated a close relatedness at the sequence level (Fig. 1a), with cluster assignments supported by pairwise average nucleotide identities between the bacteriophages (Supplementary Fig. 1). With the exception of Tortellini (P2), Xavia (P3), and ThulaThula (P5), cluster P bacteriophage genomes harbor a conserved integration-dependent immunity system, comprised of an immunity repressor flanked by a tyrosine integrase, an excise gene, and an antirepressor (Supplementary Fig. 2) that governs the transition from the lytic to lysogenic state by binding and inactivating the lysogenic repressor (Lemire *et al.* 2011; Kim and Ryu 2013). It has previously been hypothesized that conserved integration-dependent immunity systems form the ancestral state of more complex genetic switches (Broussard and Hatfull 2013), such as those present in λ bacteriophages (Oppenheim *et al.* 2005). Interestingly, a neighbor-joining tree generated from whole-genome sequences of 16 cluster G1, I1, and N bacteriophages containing an integration-dependent immunity system (Supplementary Table 2) places cluster P4–P6 bacteriophages as sister taxa to the G1, I1, and N subclusters (Fig. 1b)—a tree topology supported by the gene-specific tree based on the immunity repressor sequences (Fig. 1c).

**Fig. 2.**
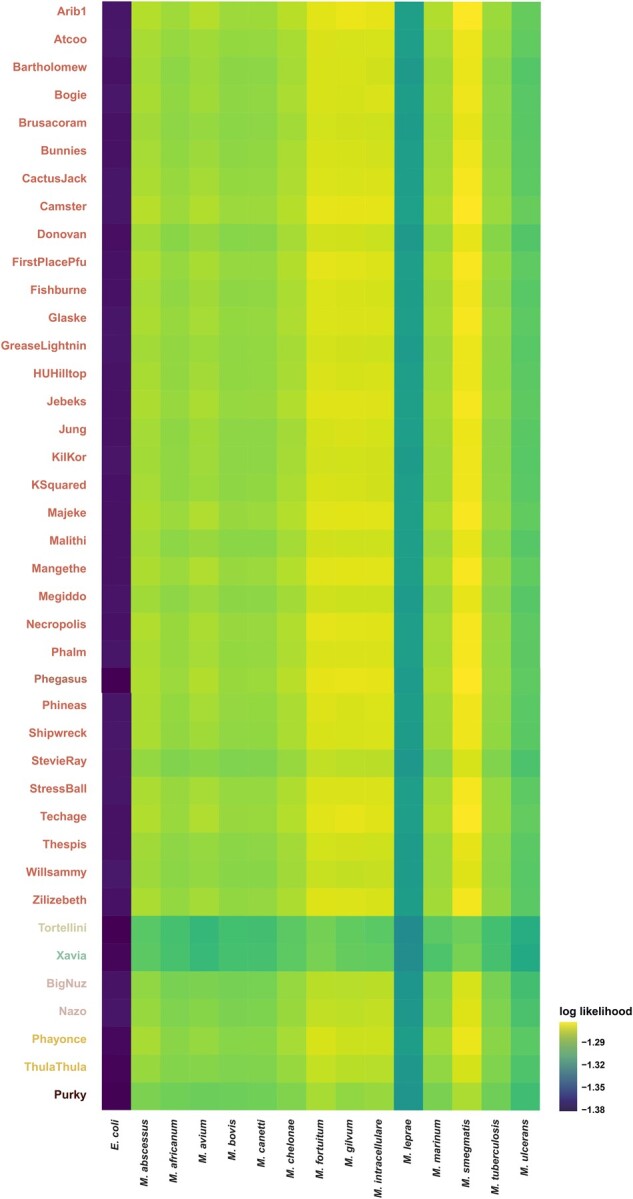
Confirmatory host range prediction. Putative bacteriophage–host interactions as predicted by WIsH (Galiez *et al.* 2017), using 40 cluster P mycobacteriophages (Supplementary Table 1), together with 14 potential bacterial hosts and *Escherichia coli* as a negative control (Supplementary Table 2). The higher the reported value, the more likely a bacteriophage is able to infect a putative host.

To explore the impact of cluster P mycobacteriophages on bacterial communities, their host ranges were computationally predicted using a combination of exploratory and confirmatory tools, together with 14 putative mycobacterial host species relevant to human health and disease. Using the exploratory method, all but 1 P1 bacteriophages (Donovan) appear restricted to the *Mycobacterium* genus (Table 1). In contrast, bacteriophages of subclusters P2–P6 are likely also able to infect the nonpathogenic microbes *Gordonia* and *Rhizobium* as well as hosts of the genera *Clostridiodes*, *Clostridium*, and *Corynebacterium*, frequently associated with human disease, including diphtheria (*Corynebacterium diphtheriae*) as well as several hospital-acquired infections (see reviews by Bernard 2012 and Mangutov *et al.* 2021). As the ability to bind to new receptors is a key step in host-range evolution (Meyer *et al.* 2012), mutations within tail protein genes might explain the predicted expanded host range of subclusters P2–P6. At the species level, confirmatory results (Fig. 2) suggest that, in addition to *M. smegmatis* mc^2^155 used to isolate the bacteriophages, subcluster P1 mycobacteriophages are likely able to infect *Mycobacterium fortuitum—*which can cause infections in the skin, lymph nodes, and joints of immunocompromised individuals (Sethi *et al.* 2014), as well as *Mycobacterium gilvum*, and *Mycobacterium intracellulare—*which can cause pulmonary infections and lymphadenitis in immunocompromised individuals (Han *et al.* 2005). In contrast, bacteriophages of subclusters P2–P6 displayed low likelihoods of infection for all tested hosts.

**Table 1.** Exploratory host range prediction.

Phage	Subcluster	*Mycobacterium*	*Gordonia*	*Clostridioides*	*Corynebacterium*	*Rhizobium*	*Clostridium*
Arib1	P1	✓					
Atcoo	P1	✓					
Bartholomew	P1	✓					
Bogie	P1	✓					
Brusacoram	P1	✓					
Bunnies	P1	✓					
CactusJack	P1	✓					
Camster	P1	✓					
Donovan	P1	✓	✓				
FirstPlacePfu	P1	✓					
Fishburne	P1	✓					
Glaske	P1	✓					
GreaseLightnin	P1	✓					
HUHilltop	P1	✓					
Jebeks	P1	✓					
Jung	P1	✓					
KilKor	P1	✓					
Ksquared	P1	✓					
Majeke	P1	✓					
Malithi	P1	✓		✓			
Mangethe	P1	✓					
Megiddo	P1	✓					
Necropolis	P1	✓					
Phalm	P1	✓					
Phegasus	P1	✓					
Phineas	P1	✓					
Shipwreck	P1	✓					
StevieRay	P1	✓					
StressBall	P1	✓					
Techage	P1	✓					
Thespis	P1	✓					
Willsammy	P1	✓					
Zilizebeth	P1	✓					
Tortellini	P2	✓	✓	✓	✓		
Xavia	P3	✓	✓	✓		✓	
BigNuz	P4	✓	✓				
Nazo	P4	✓	✓				
Phayonce	P5	✓	✓				
ThulaThula	P5	✓		✓			✓
Purky	P6	✓	✓				

Putative host genera of the 40 cluster P bacteriophages included in this study (Supplementary Table 1) as predicted by PHERI (Baláž *et al.* 2020).

**Fig. 3.**
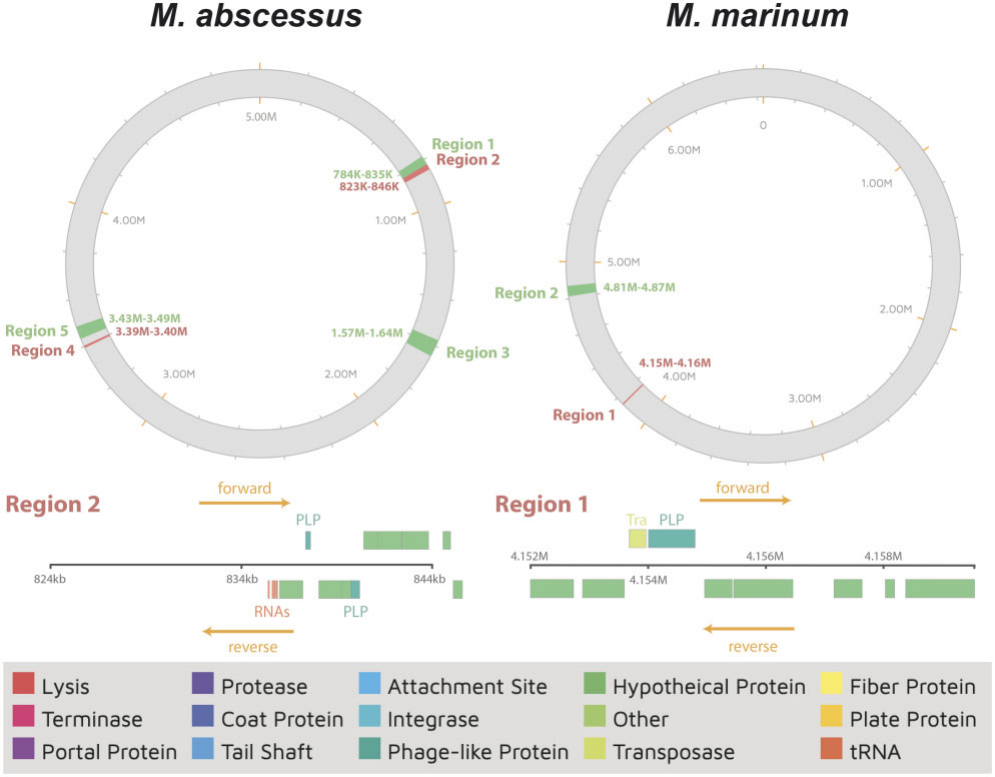
Prophage prediction. Complete (green) and incomplete (red) prophages from the integration of bacteriophages were detected in both *M. abscessus* (left) and *M. marinum* (right). Incomplete prophages from the integration of cluster P mycobacteriophages are displayed at the bottom (region 2 in *M. abscessus* and region 1 in *M. marinum*), together with the protein-coding genes contained in these regions. Phage-like proteins on forward and reverse strands (indicated by orange arrows) are displayed above and below the ruler for each region, respectively.

To investigate the temperate nature of cluster P mycobacteriophages, prophage sequences were computationally predicted within the putative host genomes. Three putative hosts (*Mycobacterium abscessus*, *Mycobacterium marinum*, and *M. smegmatis*) contain intact prophages—however, none of them correspond to prophages that stem from the integration of cluster P mycobacteriophages. In addition, incomplete prophages from the integration of cluster P mycobacteriophages were detected in both *M. abscessus* and *M. marinum* (Fig. 3)—2 opportunistic pathogens known to inflict pulmonary (Winthrop and Roy 2020) and cutaneous (Aubry *et al.* 2000) infections in humans—indicating that these hosts are at risk of incorporating virulence factors from these bacteriophages. Interestingly, the 2 partial prophages within *M. abscessus* and *M. marinum* were predicted to stem from the integration of 2 (out of only 3) cluster P bacteriophages that lack an integration-dependent immunity system (ThulaThula and Xavia, respectively).

For temperate bacteriophages, the risk of transfer of virulence factors depends (at least in part) on the presence of an *attP* region in the bacteriophage as well as a corresponding *attB* attachment site in the host genome (Pham *et al.* 2007). Putative *attP* sites in cluster P bacteriophages are similar in length to those previously reported in other mycobacteriophages (Pham *et al.* 2007; Morris *et al.* 2008) and the lack of arm-type integrase binding sites flanking the *attP* common core—known to be present in nonintegration-dependent immunity system bacteriophages such as λ (Landy 1989) and L5 (Peña *et al.* 1997) but notably absent in integration-dependent immunity system bacteriophages (Broussard *et al.* 2013)—is further evidence of a functional integration-dependent immunity system in these bacteriophages. To identify putative attachment sites, *attP* sites were compared against the genomes of 14 mycobacteria. Out of the 14 mycobacterium species tested, only 3 (*M. smegmatis*, *Mycobacterium chelonae*, and *Mycobacterium leprae*) contained a homologous *attB* bacterial attachment site, overlapping with the 3′-end of a tRNA^Thr^ gene (Supplementary Table 4), indicating that these hosts are at risk of incorporating virulence factors from bacteriophages that utilize tyrosine integrases in their integration-dependent immunity systems. Yet, despite the presence of an *attB* attachment site, 2 out of these 3 species (*M. chelonae* and *M. leprae*) were not predicted as potential hosts for any cluster P bacteriophage. However, it is important to note that WIsH evaluates host likelihood on the basis of oligonucleotide frequency similarity between the virus and host genomes. Consequently, more sophisticated approaches that rely on several distinct genomic features to predict the success of phage infection (such as advanced machine learning-based methods) may be able to provide a more complete picture of the putative host ranges.

Taken together, our computational predictions indicate that cluster P bacteriophages harboring a conserved integration-dependent immunity system likely exhibit similar host ranges. An important future endeavor will be the experimental validation of the presented computational results by phenotypic studies in order to lend further credence to the hypothesis that the type of genetic switch used to induce lysogeny plays an important role in host range evolution.

## Supplementary Material

jkac244_Supplemental_Figures

jkac244_Supplemental_Tables

## Data Availability

Genomic data for all 40 cluster P mycobacteriophages, 16 cluster G1, I1, and N bacteriophages with a previously identified integration-dependent immunity system, and 14 putative bacterial host species can be downloaded from the NCBI Sequence Read Archive using the accession numbers provided in Supplementary Tables 1–3, respectively. Supplementary Table 4 lists the mycobacteriophage integration systems and putative integration sites of cluster P mycobacteriophages in *M. chelonae*, *M. leprae*, and *M. smegmatis.*Supplementary Fig. 1 displays the pairwise average nucleotide identities of the 40 cluster P bacteriophages. Supplementary Fig. 2 displays the Phamerator map of the regions encoding the tyrosine integrase, immunity repressor, and excise genes in cluster P mycobacteriophages. Supplemental material is available at G3 online.
