# Supplementary material for: Phylogenomic analyses and host range prediction of cluster P mycobacteriophages
Source: G3 (Bethesda). 2022 Sep 12;12(11):jkac244. doi: 10.1093/g3journal/jkac244 (PMC9635641; doi:10.1093/g3journal/jkac244)
Supplement: jkac244_Supplemental_Figures [file jkac244_supplemental_figures.pdf]

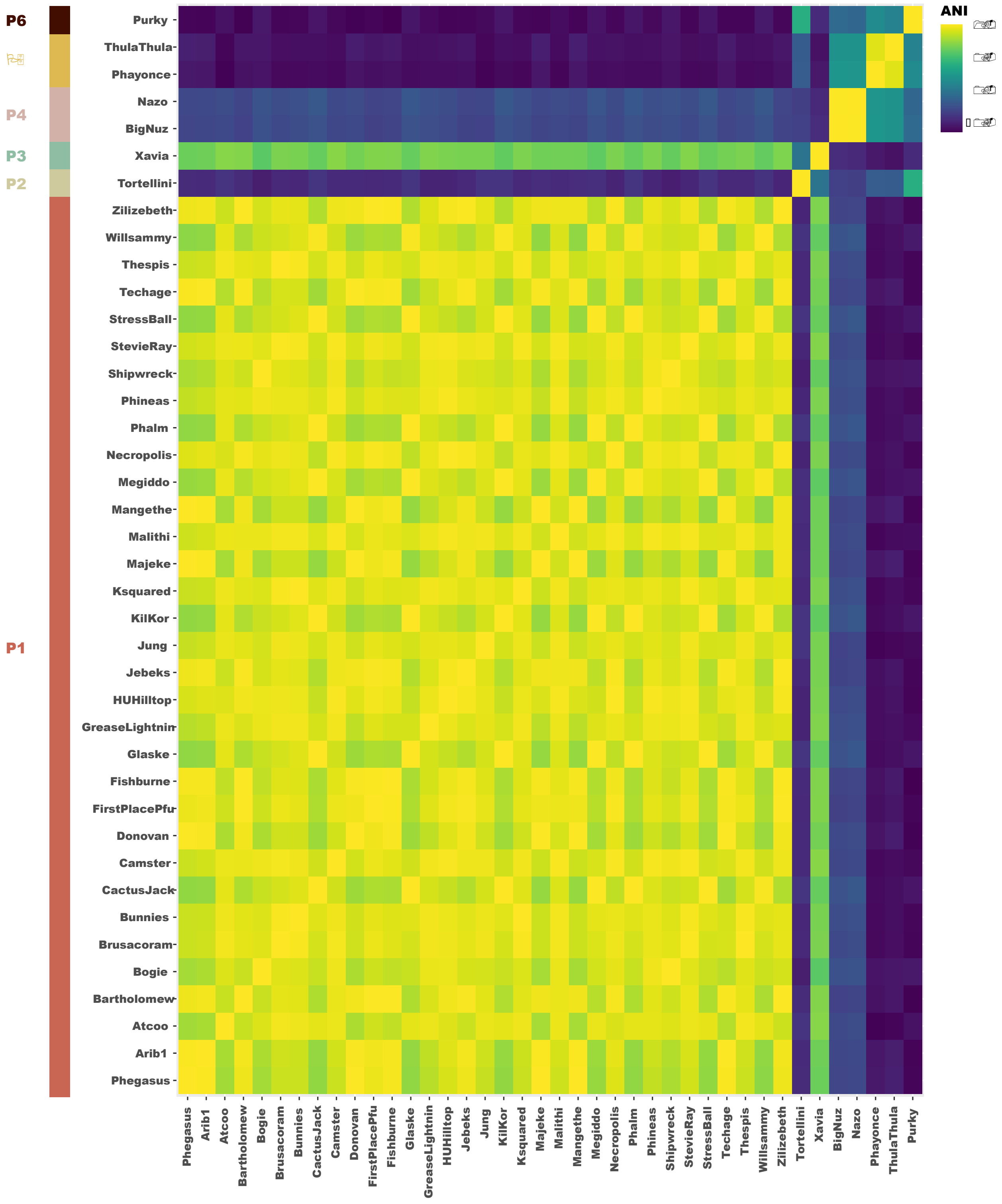

Figure S1. Heatmap of average nucleotide identity (ANI) values of 40 cluster P mycobacteriophage genomes (Table S1). Colors highlight membership in sub-clusters P1 to P6.

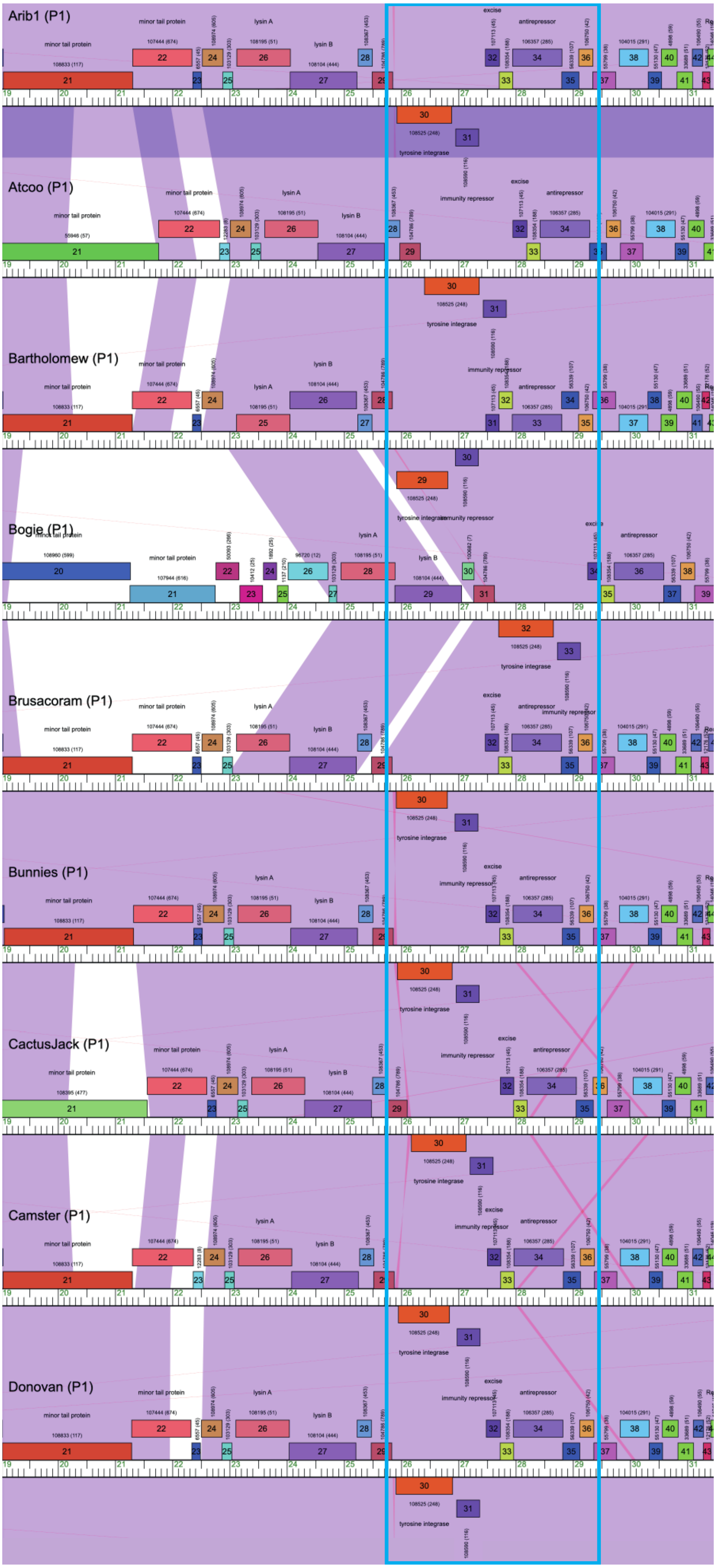
Arib1 (P1)
Atcoo (P1)
Bartholomew (P1)
Bogie (P1)
Brusacoram (P1)
Bunnies (P1)
CactusJack (P1)
Camster (P1)
Donovan (P1)
minor tail protein
lysin A
lysin B
excise
antirepressor
immunity repressor
tyrosine integrase

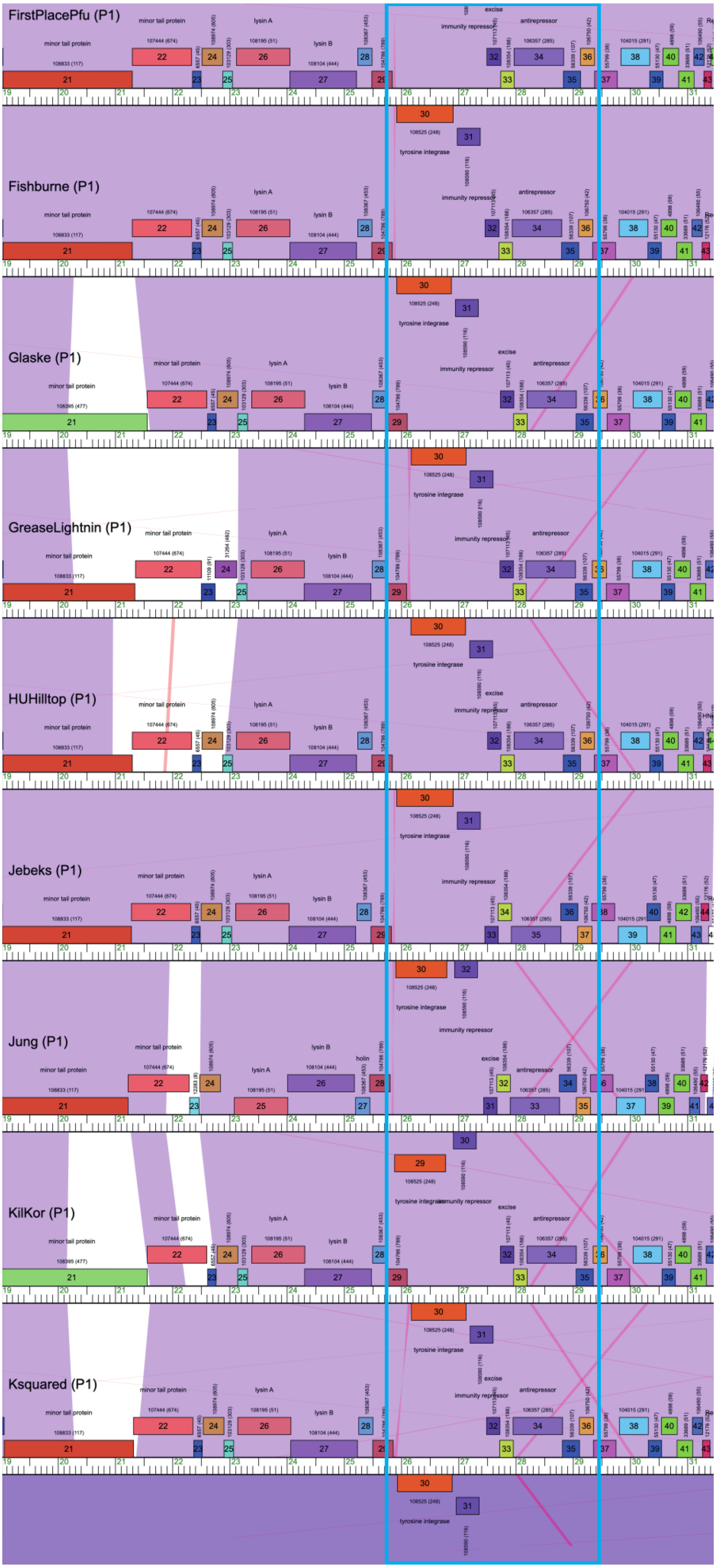

FirstPlacePfu (P1)
Fishburne (P1)
Glaske (P1)
GreaseLightnin (P1)
HUHilltop (P1)
Jebeks (P1)
Jung (P1)
KilKor (P1)
Ksquared (P1)
minor tail protein
lysin A
lysin B
holin
excise
immunity repressor
antirepressor
tyrosine integrase

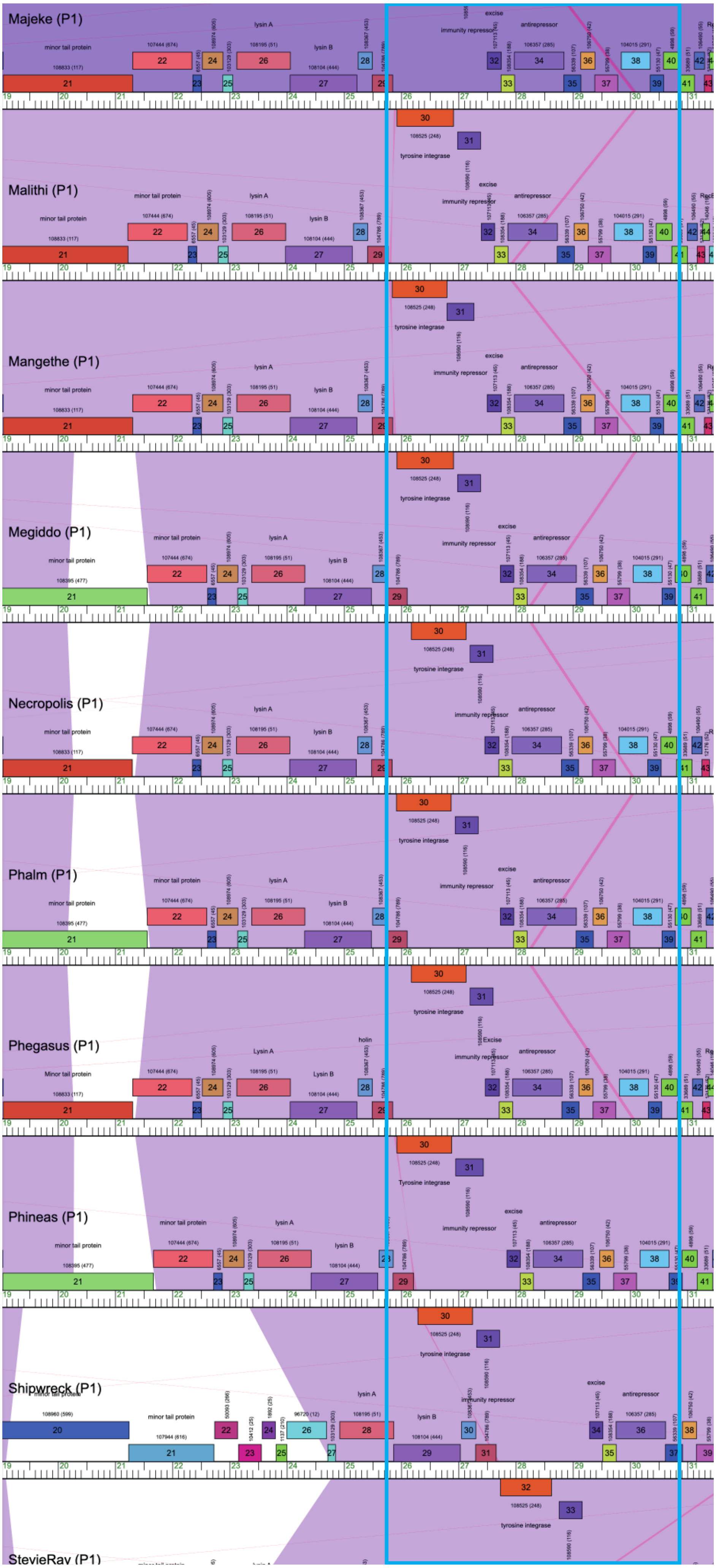

Majeke (P1)
Malithi (P1)
Mangethe (P1)
Megiddo (P1)
Necropolis (P1)
Phalm (P1)
Phegasus (P1)
Phineas (P1)
Shipwreck (P1)
StevieRay (P1)
minor tail protein
lysin A
lysin B
tyrosine integrase
immunity repressor
excise
antirepressor

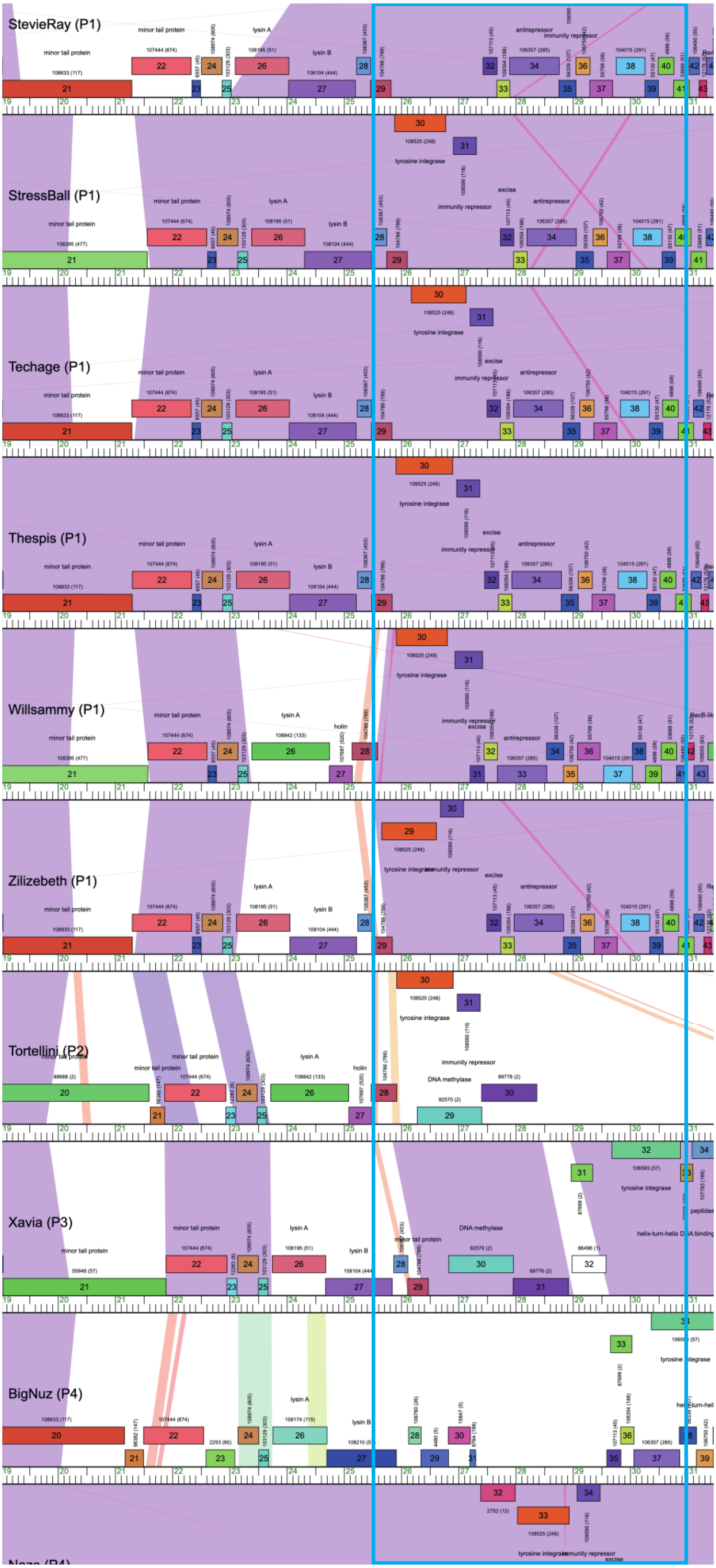

StevieRay (P1)
StressBall (P1)
Techage (P1)
Thespis (P1)
Willsammy (P1)
Zilizebeth (P1)
Tortellini (P2)
Xavia (P3)
BigNuz (P4)
minor tail protein
lysin A
lysin B
holin
antirepressor
immunity repressor
excise
tyrosine integrase
DNA methylase
helix-turn-helix DNA binding

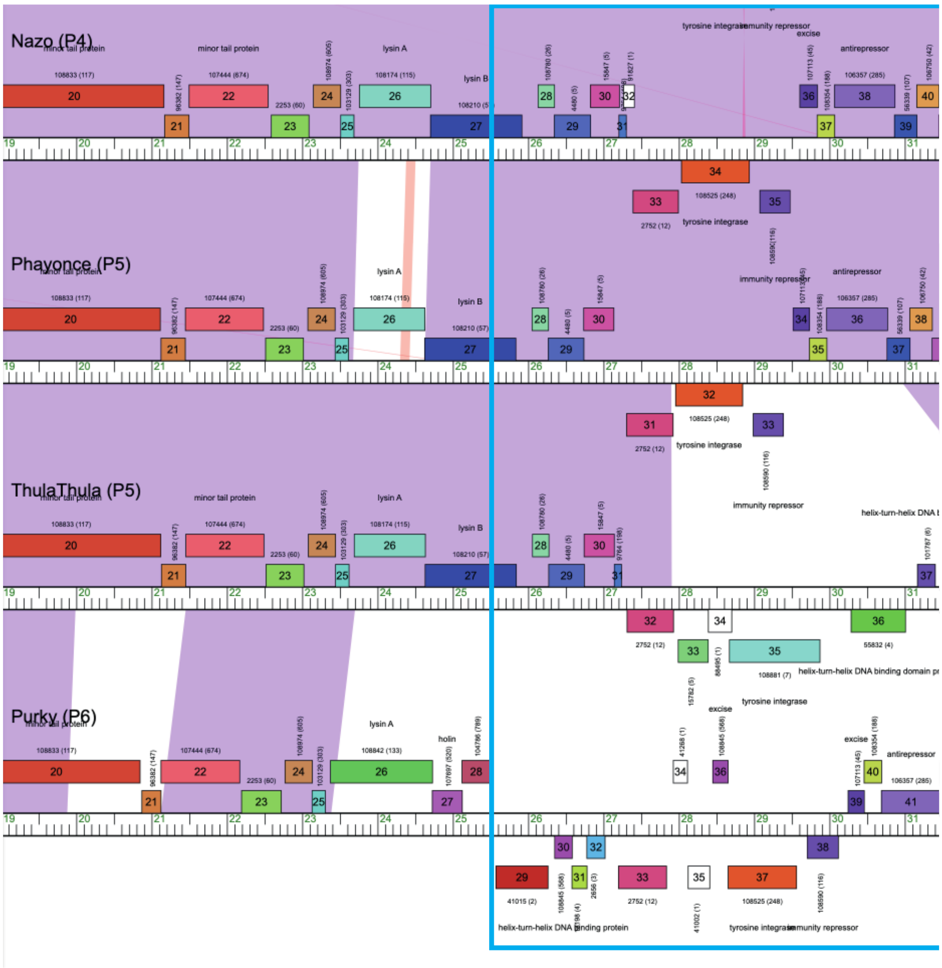

Figure S2. Phamerator map of regions encoding a tyrosine integrase, immunity repressor, and excise gene in cluster P mycobacteriophages (Table S2). All but three cluster P mycobacteriophages – Tortellini (P2), Xavia (P3), and ThulaThula (P5) – exhibit a conserved integration-dependent immunity system. In this Phamerator map, protein-coding genes with their putative functional assignments (if available) are displayed above or below a ruler, signifying genes on forward or reverse strands, respectively. The numbers shown above each gene indicate the protein family (pham) and, in parenthesis, the number of members in the pham family. Coloring between genomes represents nucleotide similarity with areas of highest similarity shown in purple (BLAST e-value = 0), followed by red (BLAST e-value of ~10-4) and white (no significant similarity). The location of the genes of interest is indicated by a blue box.
