# Supplementary material for: Phylogenomic analyses and host range prediction of cluster P mycobacteriophages
Source: G3 (Bethesda). 2022 Sep 12;12(11):jkac244. doi: 10.1093/g3journal/jkac244 (PMC9635641; doi:10.1093/g3journal/jkac244)
Supplement: jkac244_Supplemental_Tables [file jkac244_supplemental_tables.pdf]

Table S1. *Mycobacterium* cluster P bacteriophages included in the comparative analyses. Bacteriophages for which integration-dependent immunity systems had previously been identified through the computational identification of an *attP* site within the repressor gene are highlighted in blue.

Phage	Subcluster	Length (bp)	GC-content	# ORFs	# tRNAs	IDIS*	Accession #	Reference
Arib1	P1	46,732	67.5%	78	0	yes	NC_051736.1	unpublished
Atcoo	P1	49,075	67.0%	78	0	yes	NC_051729.1	unpublished
Bartholomew	P1	46,484	67.2%	77	0	yes	NC_051734.1	Doyle *et al.* 2018
Bogie	P1	48,639	66.9%	81	0	yes	MF133446.1	Doyle *et al.* 2018
Brusacoram	P1	47,618	67.0%	78	0	yes	NC_028747.1	Hatfull *et al.* 2016
Bunnies	P1	48,822	67.1%	81	1	yes	MN096356.1	unpublished
CactusJack	P1	48,222	67.3%	79	0	yes	MN892484.1	unpublished
Camster	P1	47,149	67.2%	80	0	yes	MW055902.1	unpublished
Donovan	P1	47,162	67.2%	78	0	yes	KF841477.1	Pope *et al.* 2015
FirstPlacePfu	P1	45,680	67.3%	82	0	yes	NC_051735.1	unpublished
Fishburne	P1	47,109	67.3%	77	0	yes	NC_021302.1	Hatfull *et al.* 2013
Glaske	P1	48,222	67.3%	78	0	yes	MN807250.1	unpublished
GreaseLightnin	P1	48,424	67.1%	80	0	yes	NC_051731.1	unpublished
HUHilltop	P1	46,896	67.2%	81	0	yes	MN010757.1	Pope *et al.* 2015
Jebeks	P1	45,580	67.3%	77	0	yes	NC_041969.1	Pope *et al.* 2015
Jung	P1	46,561	67.1%	77	0	yes	NC_051730.1	Van *et al.* 2020
KilKor	P1	48,916	67.2%	79	0	yes	NC_053209.1	unpublished
Ksquared	P1	48,699	67.1%	80	0	yes	NC_051732.1	Doyle *et al.* 2018
Majeke	P1	47,612	67.4%	81	0	yes	NC_051737.1	unpublished
Malithi	P1	46,870	67.1%	79	0	yes	KP027200.1	Pope *et al.* 2015
Mangethe	P1	47,612	67.4%	81	1	yes	MK016499.1	unpublished
Megiddo	P1	48,783	67.1%	78	0	yes	NC_051728.1	unpublished
Necropolis	P1	46,263	62.9%	76	0	yes	MK937604.1	unpublished
Phalm	P1	48,213	67.3%	79	0	yes	MN807248.1	unpublished
Phegasus	P1	47,578	67.4%	81	0	yes	ON637760	Howell, Versoza *et al.* 2022
Phineas	P1	47,229	67.2%	77	0	yes	NC_051733.1	Pope *et al.* 2015
Shipwreck	P1	48,670	66.9%	81	0	yes	NC_031261.1	Pope *et al.* 2015
StevieRay	P1	48,815	66.9%	81	1	yes	MF373843.1	unpublished
StressBall	P1	47,915	67.3%	78	0	yes	MN908683.1	unpublished
Techage	P1	47,094	67.4%	79	0	yes	MK919480.1	unpublished
Thespis	P1	47,618	67.0%	78	0	yes	MG198785.1	Bushhouse *et al.* 2017
Willsammy	P1	48,399	67.0%	80	0	yes	NC_051727.1	unpublished
Zilizebeth	P1	48,056	67.3%	83	1	yes	MK524508.1	unpublished
Tortellini	P2	49,658	65.8%	76	0	no	NC_041888.1	Doyle *et al.* 2018
Xavia	P3	49,808	65.9%	71	0	no	NC_051740.1	unpublished
BigNuz	P4	48,984	66.7%	82	0	yes	NC_023692.1	Pope *et al.* 2015
Nazo	P4	48,870	66.8%	83	0	yes	KX641262	unpublished
Phayonce	P5	49,203	66.7%	77	0	yes	KR080195	Pope *et al.* 2015
ThulaThula	P5	50,415	66.5%	80	0	no	MN234172	Wada *et al.* 2017
Purky	P6	50,513	66.4%	84	0	yes	MN096355.1	Pope *et al.* 2015

* integration-dependent immunity system, comprised of an immunity repressor flanked by an integrase, an excise gene, and an anti-repressor

Table S2. Bacteriophages included in the comparative analyses for which integration-dependent immunity systems had previously been identified (green: experimentally validated; blue: computationally predicted).

Phage Name	Subcluster	Length (bp)	GC-content	# ORFs	# tRNAs	Accession #	Reference
BPs	G1	41,901	66.6%	63	0	EU568876	Sampson *et al.* 2009
Cedarsite	G1	41,901	66.6%	63	0	KT355472	Hatfull *et al.* 2016
Halo	G1	42,289	66.7%	64	0	NC_008202.2	Sampson *et al.* 2009
Island3	I1	47,287	66.8%	76	0	HM152765.1	Pope *et al.* 2011
Babsiella	I1	48,420	67.1%	78	0	NC_023697.1	Hatfull *et al.* 2012
Brujita	I1	47,057	66.8%	74	0	NC_011291.1	Hatfull *et al.* 2010
Charcharodon	N	43,680	66.2%	71	0	KM588359	Hatfull *et al.* 2016
Charlie	N	43,036	66.3%	69	0	NC_023729.1	Hatfull *et al.* 2012
MichelleMyBell	N	42,240	66.0%	70	0	KF986246	Hatfull *et al.* 2016
Panchino	N	43,516	65.9%	66	0	KU935727	Hatfull *et al.* 2016
Phrann	N	44,872	66.3%	67	0	KU935731	Hatfull *et al.* 2016
Pipsqueaks	N	43,679	66.3%	73	0	KU935730	Hatfull *et al.* 2016
Redi	N	42,594	66.1%	70	0	NC_023730.1	Hatfull *et al.* 2012
SkinnyPete	N	43,478	66.4%	67	0	KU935729	Hatfull *et al.* 2016
Xeno	N	42,395	66.8%	69	0	KU935728	Hatfull *et al.* 2016
Xerxes	N	43,698	66.3%	72	0	KU935726	Hatfull *et al.* 2016

Table S3. *Mycobacteria* included in the comparative analyses.

Mycobacterium	# Genes	Length (kb)	GC-content	Accession #	Reference
M. abscessus	4,957	4,618	64.2%	CP004374	Kim *et al.* 2013
M. africanum	4,069	4,493	65.1%	CP014617	Hurtado *et al.* 2016
M. avium	3,935	3,981	69.3%	AE016958	Li *et al.* 2005
M. bovis	3,952	3,972	65.6%	AM408590	Brosch *et al.* 2007
M. canetti	4,139	4,482	65.6%	HE572590	Bentley *et al.* 2012
M. chelonae	4,943	5,061	64.0%	CP050145	Gu *et al.* 2020
M. fortuitum	6,023	6,255	66.2%	CP011269	Costa *et al.* 2015
M. gilvum	5,139	5,077	67.9%	CP002385	Kallimanis *et al.* 2011
M. intracellulare	5,143	4,936	68.1%	CP003322	Kim *et al.* 2012
M. leprae	1,604	1,620	57.8%	AL450380	Cole *et al.* 2001
M. marinum	5,422	5,973	65.2%	CP000854	Stinear *et al.* 2008
M. smegmatis	6,692	6,508	67.4%	CP001663	Deshayes *et al.* 2007
M. tuberculosis	3,935	3,981	65.6%	AL123456	Cole *et al.* 1998
M. ulcerans	4,159	4,074	65.4%	CP000325	Stinear *et al.* 2007

Table S4. Mycobacteriophage integration systems and putative integration sites of *Mycobacterium* cluster P bacteriophages in *M. chelonae* Myco3a (*attB* location: tRNA^{Thr} ; 447,412–447,737 bp), *M. leprae* TN (*attB* location: tRNA^{Thr} ; 271,936–271,975 bp), and *M. smegmatis* mc^2 (*attB* location: tRNA^{Thr} ; 6,222,599–6,222,637 bp).

	BLASTn percent identity		
**Phage**	***M. chelonae***	***M. leprae***	***M. smegmatis***
Arib1	38/40 (95%)	40/40 (100%)	39/39 (100%)
Atcoo	38/40 (95%)	40/40 (100%)	41/42 (98%)
Bartholomew	38/40 (95%)	40/40 (100%)	39/39 (100%)
Bogie	38/40 (95%)	40/40 (100%)	39/39 (100%)
Brusacoram	38/40 (95%)	40/40 (100%)	41/42 (98%)
Bunnies	38/40 (95%)	40/40 (100%)	41/42 (98%)
CactusJack	38/40 (95%)	40/40 (100%)	41/42 (98%)
Camster	38/40 (95%)	40/40 (100%)	41/42 (98%)
Donovan	38/40 (95%)	40/40 (100%)	39/39 (100%)
FirstPlacePfu	38/40 (95%)	40/40 (100%)	39/39 (100%)
Fishburne	38/40 (95%)	40/40 (100%)	39/39 (100%)
Glaske	38/40 (95%)	40/40 (100%)	41/42 (98%)
GreaseLightnin	38/40 (95%)	40/40 (100%)	41/42 (98%)
HUHilltop	38/40 (95%)	40/40 (100%)	39/39 (100%)
Jebeks	38/40 (95%)	40/40 (100%)	39/39 (100%)
Jung	38/40 (95%)	40/40 (100%)	41/42 (98%)
KilKor	38/40 (95%)	40/40 (100%)	41/42 (98%)
Ksquared	38/40 (95%)	40/40 (100%)	41/42 (98%)
Majeke	38/40 (95%)	40/40 (100%)	39/39 (100%)
Malithi	38/40 (95%)	40/40 (100%)	41/42 (98%)
Mangethe	38/40 (95%)	40/40 (100%)	39/39 (100%)
Megiddo	38/40 (95%)	40/40 (100%)	41/42 (98%)
Necropolis	38/40 (95%)	40/40 (100%)	39/39 (100%)
Phalm	38/40 (95%)	40/40 (100%)	41/42 (98%)
Phegasus	38/40 (95%)	40/40 (100%)	39/39 (100%)
Phineas	38/40 (95%)	40/40 (100%)	39/39 (100%)
Shipwreck	38/40 (95%)	40/40 (100%)	39/39 (100%)
StevieRay	38/40 (95%)	40/40 (100%)	41/42 (98%)
StressBall	38/40 (95%)	40/40 (100%)	41/42 (98%)
Techage	38/40 (95%)	40/40 (100%)	39/39 (100%)
Thespis	38/40 (95%)	40/40 (100%)	41/42 (98%)
Willsammy	38/40 (95%)	40/40 (100%)	41/42 (98%)
Zilizebeth	38/40 (95%)	40/40 (100%)	39/39 (100%)
BigNuz	38/40 (95%)	40/40 (100%)	39/39 (100%)
Nazo	38/40 (95%)	40/40 (100%)	39/39 (100%)
Phayonce	42/45 (93%)	44/45 (98%)	43/44 (98%)
Purky	39/41 (95%)	41/41 (100%)	42/43 (98%)

REFERENCES

Bentley SD, Comas I, Bryant JM, Walker D, Smith NH, et al. 2012. The genome of *Mycobacterium africanum* West African 2 reveals a lineage-specific locus and genome erosion common to the *M. tuberculosis* complex. *PLoS Negl. Trop. Dis.* 6(2): e1552.

Brosch R, Gordon SV, Garnier T, Eiglmeier K, Frigui W, et al. 2007. Genome plasticity of BCG and impact on vaccine efficacy. *Proc. Natl. Acad. Sci. U. S. A.* 104(13): 5596–5601.

Bushhouse DZ, Bowen EK, Wolyniak MJ. 2017. Isolation and genome annotation of mycobacteriophage Thespis. *H-SC Journal of Sciences*. 6.

Cole ST, Eiglmeier K, Parkhill J, James KD, Thomson NR, et al. 2001. Massive gene decay in the leprosy bacillus. *Nature*. 409(6823): 1007–1011.

Cole ST, Brosch R, Parkhill J, Garnier T, Churcher C, et al. 1998. Deciphering the biology of *Mycobacterium tuberculosis* from the complete genome sequence. *Nature*. 393(6685): 537–544.

Costa KC, Bergkessel M, Saunders S, Korlach J, Newman DK. 2015. Enzymatic degradation of phenazines can generate energy and protect sensitive organisms from toxicity. *mBio*. 6(6): e01520-15.

Deshayes C, Perrodou E, Gallien S, Euphrasie D, Schaeffer C, et al. 2007. Interrupted coding sequences in *Mycobacterium smegmatis*: authentic mutations or sequencing errors? *Genome Biol.* 8(2): R20.

Doyle EL, Fillman CL, Reyna NS, Tobiason DM, Westholm DE, et al. 2018. Genome sequences of four cluster P mycobacteriophages. *Genome Announc.* 6(2): e01101-17.

Gu CH, Zhao C, Hofstaedter C, Tebas P, Glaser L, et al. 2020. Investigating hospital *Mycobacterium chelonae* infection using whole genome sequencing and hybrid assembly. *PLoS One*. 15(11): e0236533.

Hatfull GF, Jacobs-Sera D, Lawrence JG, Pope WH, Russell DA, et al. 2010. Comparative genomic analysis of 60 mycobacteriophage genomes: genome clustering, gene acquisition, and gene size. *J. Mol. Biol.* 397(1): 119–143.

Hatfull GF, Science Education Alliance Phage Hunters Advancing Genomics and Evolutionary Science (SEA-PHAGES) Program, KwaZulu-Natal Research Institute for Tuberculosis and HIV (K-RITH) Mycobacterial Genetics Course, University of California – Los Angeles Research Immersion Laboratory in Virology, Phage Hunters Integrating Research and Education (PHIRE) Program. 2016. Complete genome sequences of 61 mycobacteriophages. *Genome Announc*. 4(4): e00389-16.

Hatfull GF, Science Education Alliance Phage Hunters Advancing Genomics and Evolutionary Science (SEA-PHAGES) Program, KwaZulu-Natal Research Institute for Tuberculosis and HIV (K-RITH) Mycobacterial Genetics Course, University of California – Los Angeles Research Immersion Laboratory in Virology, Phage Hunters Integrating Research and Education (PHIRE) Program. 2013. Complete genome sequences of 63 mycobacteriophages. *Genome Announc*. 1(6): e00847-13.

Hatfull GF, Science Education Alliance Phage Hunters Advancing Genomics and Evolutionary Science Program, KwaZulu-Natal Research Institute for Tuberculosis and HIV Mycobacterial Genetics Course Students, Phage Hunters Integrating Research and Education Program. 2012. Complete genome sequences of 138 mycobacteriophages. *J. Virol.* 86(4): 2382-4.

Howell* AA, Versoza* CJ, Cerna G, Johnston T, Kakde S, Karuku K, Kowal M, Monahan J, Murray J, Nguyen T, Sanchez Carreon A, Song E, Streiff A, Su B, Youkhana F, Munig S, Patel Z, So M, Sy M, Weiss S, Zhou Y, Pfeifer SP. 2022. Complete genome sequence of the cluster P mycobacteriophage Phegasus. *Microbiol. Resour. Announc.* e00540-22.

Hurtado UA, Solano JS, Rodriguez A, Robledo J, Rouzaud F. 2016. Draft genome sequence of a *Mycobacterium africanum* clinical isolate from Antioquia, Colombia. *Genome Announc*. 4(3): e00486-16.

Kallimanis A, Karabika E, Mavromatis K, Lapidus A, Labutti KM, et al. 2011. Complete genome sequence of *Mycobacterium sp*. strain (Spyr1) and reclassification to *Mycobacterium gilvum* Spyr1. *Stand Genomic Sci*. 5(1): 144–153.

Kim BJ, Kim BR, Hong SH, Seok SH, Kook YH, et al. 2013. Complete genome sequence of *Mycobacterium massiliense* clinical strain Asan 50594, belonging to the type II genotype. *Genome Announc*. 1(4): e00429-13.

Kim BJ, Choi BS, Lim JS, Choi IY, Lee JH, et al. 2012. Complete genome sequence of *Mycobacterium intracellulare* strain ATCC 13950(T). *J. Bacteriol*. 194(10): 2750.

Li L, Bannantine JP, Zhang Q, Amonsin A, May BJ, et al. 2005. The complete genome sequence of *Mycobacterium avium* subspecies paratuberculosis. *Proc. Natl. Acad. Sci. U. S. A*. 102(35): 12344–12349.

Pope WH, Bowman CA, Russell DA, Jacobs-Sera D, Asai DJ, et al. 2015. Whole genome comparison of a large collection of mycobacteriophages reveals a continuum of phage genetic diversity. *eLife*. 4: e06416.

Pope WH, Jacobs-Sera D, Russell DA, Peebles CL, Al-Atrache Z, et al. 2011. Expanding the diversity of mycobacteriophages: insights into genome architecture and evolution. *PLoS One*. 6(1): e16329.

Pope WH, Jacobetz E, Johnson CA, Kihle BL, Sobeski MA, et al. 2015. Genome sequence of mycobacteriophage Phayonce. *Genome Announc*. 3(3): e00598-15.

Sampson T, Broussard GW, Marinelli LJ, Jacobs-Sera D, Ray M, et al. 2009. Mycobacteriophages BPs, Angel and Halo: comparative genomics reveals a novel class of ultra-small mobile genetic elements. *Microbiology*. 155(Pt 9): 2962.

Stinear TP, Seemann T, Harrison PF, Jenkin GA, Davies JK, et al. 2008. Insights from the complete genome sequence of *Mycobacterium marinum* on the evolution of *Mycobacterium tuberculosis*. *Genome Res*. 18(5): 729–741.

Stinear TP, Seemann T, Pidot S, Frigui W, Reysset G, et al. 2007. Reductive evolution and niche adaptation inferred from the genome of *Mycobacterium ulcerans*, the causative agent of Buruli ulcer. *Genome Res*. 17(2): 192–200.

Van R, Nie W, Abdela F, Eivazi B, Kickbusch D, et al. 2020. Complete genome sequences of cluster P1 and cluster C1 *Mycobacterium smegmatis* phages Jung and Ronan. *Microbiol. Resour. Announc*. 9(34): e00678-20.

Wada T, Hijikata M, Maeda S, Hang NTL, Thuong PH, et al. 2017. Complete genome sequence of a *Mycobacterium tuberculosis* strain belonging to the east African-Indian family in the indo-oceanic lineage, isolated in Hanoi, Vietnam. *Genome Announc*. 5(24): e00509-17.
